# Foxg1 is required to limit the formation of ciliary margin tissue and Wnt/β-catenin signalling in the developing nasal retina of the mouse^☆^

**DOI:** 10.1016/j.ydbio.2013.04.017

**Published:** 2013-08-15

**Authors:** Vassiliki Fotaki, Rowena Smith, Thomas Pratt, David J. Price

**Affiliations:** University of Edinburgh, Centre for Integrative Physiology, Hugh Robson Building, George Square, Edinburgh EH8 9XD, UK

**Keywords:** Foxg1, Wnt/β-catenin signalling, Eye development, Ciliary margin, Mouse

## Abstract

The ciliary margin (CM) develops in the peripheral retina and gives rise to the iris and the ciliary body. The Wnt/β-catenin signalling pathway has been implicated in ciliary margin development. Here, we tested the hypothesis that in the developing mouse retina Foxg1 is responsible for suppressing the Wnt/β-catenin pathway and restricting CM development. We showed that there is excess CM tissue in *Foxg1*^−*/*−^ null embryos and this expansion is more pronounced in the nasal retina where *Foxg1* normally shows its highest expression levels. Results on expression of a reporter allele for Wnt/β-catenin signalling and of *Lef1*, a target of Wnt/β-catenin signalling, displayed significant upregulation of this pathway in *Foxg1*^−*/*−^ nulls at embryonic days 12.5 and 14.5. Interestingly, this upregulation was observed specifically in the nasal retina, where normally very few Wnt-responsive cells are observed. These results indicate a suppressive role of Foxg1 on this signalling pathway. Our results reveal a new role of Foxg1 in limiting CM development in the nasal peripheral retina and add a new molecular player in the developmental network involved in CM specification.

## Highlights

• *Foxg1* is expressed in a nasal-high to temporal-low gradient in developing retina.
• Ciliary margin expansion is observed nasally in the *Foxg1*^−*/*−^ mutant retina.
• Wnt/β-catenin signalling is upregulated in the *Foxg1*^−*/*−^ peripheral retina nasally.
• A new role of Foxg1 in controlling ciliary margin development is proposed.

## Introduction

During eye development the optic cup gives rise to the centrally located neural retina (NR), an outer located layer known as retinal pigment epithelium (RPE) and to the peripherally located ciliary margin (CM), which lies at the interface between the NR and the RPE. Although of neural origin, the CM generates two non-neural epithelial eye structures: the ciliary body (CB) proximally, which is responsible for the production of the aqueous humor and maintenance of the intraocular pressure, and the iris distally, whose role is to control the amount of light entering the eye and the circulation of the aqueous humor (reviewed in Davis-Silberman and Ashery-Padan, 2008). The study of the molecular properties of the CM has drawn a lot of attention, in part because mutations in genes responsible for its formation are associated with an increased risk of glaucoma (Gould et al., 2004; Hannenhalli and Kaestner, 2009).

Several pieces of evidence have implicated the Wnt/β-catenin signalling pathway in CM development in both chicks and mice (Cho and Cepko, 2006; Esteve et al., 2011; Fuhrmann et al., 2009; Liu et al., 2003, 2006, 2007). Among the multiple members of the Wnt family of morphogens, *Wnt2b* shows a characteristic expression pattern in the CM (Jasoni et al., 1999; Kubo et al., 2003; Liu et al., 2003) and has been implicated in CM fate specification (Cho and Cepko, 2006). Reporter mice for the Wnt/β-catenin signalling pathway and chicks electroporated with a Wnt signalling reporter have revealed that this pathway is active in the CM and RPE (Cho and Cepko, 2006; Fuhrmann et al., 2009; Liu et al., 2006). In agreement with this, downstream targets of Wnt signalling such as Lef1 and Axin2 have also been shown to be expressed in these structures (Burns et al., 2008; Fuhrmann et al., 2009; Kubo et al., 2003; Liu et al., 2006). Furthermore, a mouse strain that expresses a constitutively active form of β-catenin in the peripheral retina promotes CM development at the expense of NR, while conditional inactivation of the β-catenin gene in the retina leads to disrupted development of the CM (Liu et al., 2007). These data indicate that Wnt/β-catenin signalling is required for and promotes normal CM development. So far, however, there is no information regarding the factor or factors that limit the development of this eye structure.

Foxg1 is a transcriptional repressor (Marcal et al., 2005; Yao et al., 2001) essential for mouse survival (Xuan et al., 1995). It is mainly expressed in the developing telencephalon, hypothalamus, optic chiasm and retina (Dou et al., 1999; Fotaki et al., 2006; Hatini et al., 1994; Huh et al., 1999; Marcus et al., 1999; Pratt et al., 2004; Tao and Lai, 1992; Tian et al., 2008; Xuan et al., 1995). Foxg1's role in the developing telencephalon has been studied extensively and different reports have shown that this protein is required for normal neural proliferation and differentiation (Dou et al., 1999; Hanashima et al., 2007; Hanashima et al., 2004; Manuel et al., 2011; Martynoga et al., 2005; Xuan et al., 1995). In addition, Foxg1 is essential for proper telencephalic patterning (Danesin et al., 2009; Dou et al., 1999; Manuel et al., 2010; Martynoga et al., 2005).

Less is known about the role of Foxg1 in the developing eye. Null mutants for Foxg1 (*Foxg1*^−*/*−^) have micropthalmic eyes with a large ventral coloboma (Huh et al., 1999; Xuan et al., 1995). *Foxg1*^−*/*−^ retinas display abnormal folds and lack an obvious optic stalk structure (Huh et al., 1999). However, they retain normal layering of the retina (Huh et al., 1999) and have a similar number of retinal ganglion cells (RGCs) to that of control littermates (Pratt et al., 2004; Tian et al., 2008).

Foxg1 has been implicated in diverse signalling pathways responsible for dorso-ventral telencephalic patterning including those of Tgf-β, Bmp, Wnt, Shh and Fgf (Danesin et al., 2009; Dou et al., 2000, 1999; Martynoga et al., 2005). In the zebrafish telencephalon, foxg1 has been shown to repress the Wnt/β-catenin pathway by direct binding to *wnt8b*, limiting pallial cell identities (Danesin et al., 2009).

Here, we have examined the hypothesis that in the developing mouse retina Foxg1 is responsible for suppressing the Wnt/β-catenin pathway and restricting the development of CM tissue. We show that CM tissue is expanded in *Foxg1*^−*/*−^ null embryos and this expansion is more pronounced in the nasal retina where *Foxg1* normally shows its highest expression levels. *Foxg1*^−*/*−^ nulls crossed to a reporter mouse for Wnt/β-catenin signalling display a significant upregulation of this pathway at embryonic day (E) 12.5 and E14.5. Interestingly, this upregulation is observed specifically in the nasal retina, where normally very few Wnt-responsive cells are observed. These results indicate a suppressive role of Foxg1 on this signalling pathway. Our results reveal a new role of Foxg1 in maintaining the normal size of the CM in the nasal peripheral retina and add a new molecular player in the developmental network involved in CM specification.

## Materials and methods

### Animals

Mice were maintained, bred and handled according to Home Office (UK) regulations.

The following mouse lines were used for this study: a Foxg1-lacZ heterozygous strain (Xuan et al., 1995) kept on an F1 background (CBAxC57/B6); a Foxg1-cre heterozygous strain (Hebert and McConnell, 2000) kept on an F1 background; a BAT-gal reporter strain (Maretto et al., 2003) kept on a C57/B6 background was crossed to the *Foxg1*^*+/cre*^ mouse to generate compound Foxg1-cre heterozygous/BAT-gal mutants. For the GFP expression experiment, a Gsh2-cre strain (Kessaris et al., 2006) was crossed to the *Foxg1*^*+/lacZ*^ mouse carrying a GFP reporter allele (Sousa et al., 2009). Heterozygous mice were genotyped as described in the corresponding references. They were intercrossed to generate homozygous mutants which were identified by phenotyping (*Foxg1*^−*/*−^ and *Foxg1*^−*/*−^*;GFP*^*+*^ mutants) and genotyping (*BAT-gal*^*+*^*;Foxg1*^−*/*−^ mutants).

As no differences were detected between wild types (*Foxg1*^*+/+*^) and heterozygotes (*Foxg1*^*+/*−^) these embryos were both used in the control group.

The day the vaginal plug was detected was designated as embryonic day (E) 0.5.

### Histology, immunohistochemistry, immunofluorescence

Embryos were collected and fixed in 4% paraformaldehyde in 0.1 M phosphate buffer, pH 7.4. They were processed for paraffin or cryostat embedding according to previously described protocols (Fotaki et al., 2011, 2008, 2006).

Immunohistochemistry and immunofluorescence were carried out as previously described (Fotaki et al., 2008, 2006). The following antibodies were used in this study: (A) rabbit polyclonals: β-gal (1:1000, Invitrogen, Molecular Probes); Lef1 (1:1000, Cell Signalling); Mitf (1:1000, kindly provided by Prof H. Arnheiter); Sox2 (1:3000, Millipore); Vsx2 (1:1000, kindly provided by Prof C.L. Cepko); (B) mouse monoclonals: Cyclin D1 (1:5000; ab140302 Abcam); Otx1 (1:50, DSHB); Pax6 (1:200, DSHB); (C) a goat polyclonal GFP (1:1000, ab6673 Abcam); (D) a rat monoclonal BrdU (1:200; ab6326 Abcam).

### In situ hybridisation

In situ hybridisation was performed as previously described (Wallace and Raff, 1999). Riboprobes were synthesised using the DIG-labelling system according to the manufacturer's protocol (Roche). The following mouse riboprobes were used in this study: *Bmp4*, *Tbx5* and *Vax2* (kindly provided by Dr R. Hindges); *Foxg1* and *Wnt2b* (kindly provided Dr T. Theil); *Msx1* (kindly provided by Dr L. Lettice); *Lef1* (kindly provided by Dr O. Machon) and *Sfrp2* (kindly provided by Dr A. Rattner).

### Imaging

DAB and in situ images were taken with a Leica DFC480 camera connected to a Leica DMNB epifluorescence microscope. Fluorescence images were taken with a Leica DM5500B automated upright microscope connected to a DFC360FX camera. Confocal images were taken with a Zeiss LSM510 confocal system coupled to a Coherent Mira mode-locked Ti:Sapphire multi-photon laser.

### Cell counts

The labelling index (LI) was calculated as the ratio of BrdU-positive to total number of cells on E11.5 control and mutant sections. Pregnant females were injected with 50–70 mg/kg BrdU (10 mg/ml in 0.9% NaCl, i.p.) and sacrificed 30 min later. Cell counts were performed within three 50 µm bins, placed along the peripheral retina and were the average of at least six control and four mutant 10 µm thick sections. A total of three eyes from two different specimens were used for each genotype.

A one way ANOVA was used to calculate statistical differences in the LI between bins of the same genotype. A student's t-test was used to calculate statistical differences between corresponding bins of controls and mutants. Differences were considered significant for *P* values ≤0.01.

Total numbers of β-gal-positive cells were counted along the entire retina on consecutive 10 μm thick sections 30 μm apart. A total of 6 eyes from 3 different specimens were used for each genotype.

Numbers of nasal and temporal β-gal-positive cells were counted on consecutive 10 µm thick horizontal sections 30 µm apart. The most dorsal and ventral sections, where the nasal and temporal retina could not be defined with precision, were excluded from the counts. A total of 6 eyes from 3 different specimens for controls and 4 eyes from 2 different specimens for mutants were used.

Lef1-immunopositive cells were the average from two consecutive 10 μm thick sections, 30 μm apart, counted in the nasal peripheral retina of 3 control and 3 mutant eyes.

A non-parametric test (Mann-Whitney) was used to calculate statistical differences between control and *Foxg1*^−*/*−^ mutant total, nasal, and temporal β-gal-positive cells and Lef1-positive nasal cells. Differences were considered significant for *P* values ≤0.01.

## Results

### Foxg1 retinal expression

**Fig. 1:**
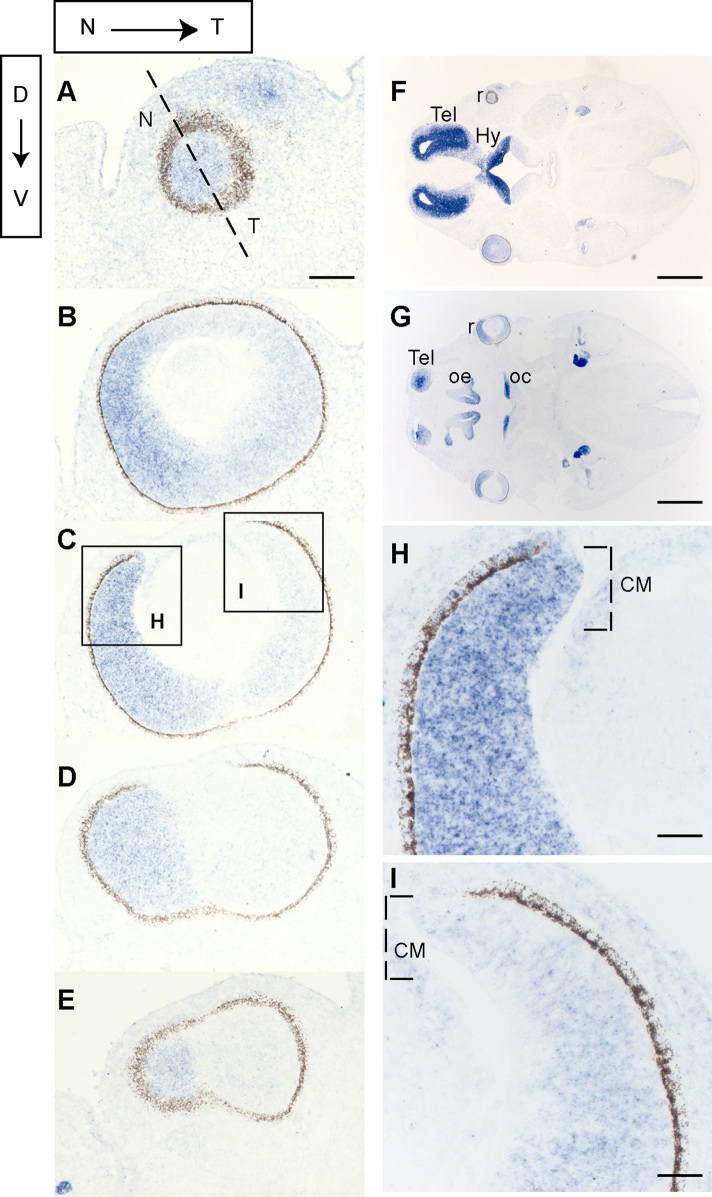
Foxg1 retinal expression is graded from ^high^nasal-to-^low^ temporal. In situ hybridisation of *Foxg1* on consecutive dorsal (D) to ventral (V) retinal E12.5 wild type sections (A–E). (F) and (G) are low power images of (A) and (C) respectively. (H) and (I) are high power images of the squared areas in (C). The dashed brackets in (H) and (I) indicate the approximate position of the ciliary margin (CM) N –> T in this and subsequent figures indicates the nasal-temporal axis. Abbreviations: Hy, hypothalamus; oc, optic chiasm; oe, olfactory epithelium; r, retina; Tel, telencephalon. Scale bars: A–E, 100 μm; F, G, 800 μm; H, I, 50 μm.

We had previously described high expression of Foxg1 in the dorsal and nasal retina at embryonic day (E) 14.5, based on beta-galactosidase (β-gal) expression in *Foxg1*^*lacZ/+*^ heterozygous embryos (Pratt et al., 2004; Tian et al., 2008). Here, we examined the retinal expression pattern of the *Foxg1* transcript by in situ hybridisation at E12.5 (Fig. 1). Our results show *Foxg1* expression in the form of a ^high^nasal-to-^low^temporal gradient (Fig. 1A–E). Also *Foxg1* is less intensely expressed in the most ventral retinal sections compared to the dorsal ones (compare Fig. 1A–C to D,E). Similar results were obtained at E14.5 (data not shown). High power images show that the strong *Foxg1* expression nasally extends to the distal edge of the retina, where the ciliary margin (CM) is located (Fig. 1H). In temporal sections, *Foxg1* expression, although detectable, was not very strong and probably does not stain all CM cells (Fig. 1I). The overall expression level of *Foxg1* in the eye was lower than that in the forebrain (compare intensity of staining in the eye with that in the optic chiasm and telencephalon in sections 1F and 1G) and this was confirmed quantitatively by RT-qPCR (data not shown).

### Morphology of the *Foxg1*^−*/*−^ retina

**Fig. 2:**
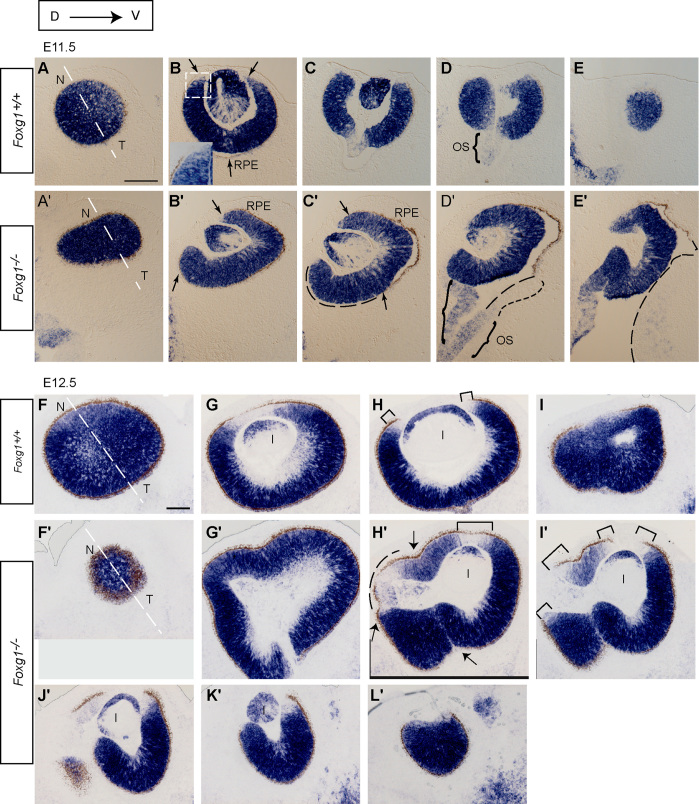
The morphology of the *Foxg1*^−*/*−^ mutant optic cup displays severe abnormalities. In situ hybridisation for *Sfrp2* in control (*Foxg1*^*+/+*^) (A–E; F–I) and mutant (*Foxg1*^−*/*−^) (A′-E′; F′-L′) on consecutive dorsal (D) to ventral (V) E11.5 (A–E′) and E12.5 (F–L′) horizontal eye sections. Arrows in (B), (B′) and (C′) indicate the retinal pigment epithelium (RPE). The dotted square in (B) demarcates the high power inset, which allows the RPE to be distinguished from the strong *Sfrp2* retinal staining. The dashed lines in (C′–E′) indicate the area of the optic cup that is not yet surrounded by RPE. Curly brackets in (D,D′) indicate the optic stalk (OS). The small-gapped dashed line in (D′) demarcates the continuation of tissue that is *Sfrp2*-negative. Brackets (H,H′,I′) demarcate *Sfrp2*-negative regions, which correspond to ciliary margin tissue. Arrows in (H′) point to the retinal pigment epithelium, which, by E12.5, surrounds the entire mutant optic cup, in both the temporal and nasal parts. Abbreviations: l, lens. Scale bar in (A) corresponds to 100 μm and applies for all panels (A–L′).

During eye development the *Foxg1*^−*/*−^ optic cup fails to close at the optic fissure leading to a large ventral coloboma and the retina elongates abnormally (Huh et al., 1999). As development progresses, the *Foxg1*^−*/*−^ retina becomes severely distorted making it difficult to interpret its phenotype. To gain insight into the gradual alterations of the *Foxg1*^−*/*−^ retina and understand its morphology, we examined horizontal sections of *Foxg1*^−*/*−^ and control littermates at E11.5 and E12.5 using in situ hybridisation for *Sfrp2*. At E11.5, *Sfrp2* labels most retinal cells both in controls and *Foxg1*^−*/*−^ mutants (Fig. 2A–E′). At this stage, both the nasal and temporal retinal pigment epithelium (RPE) have developed in controls, surrounding the entire retina (Fig. 2A–E). In *Foxg1*^−*/*−^ mutants, the RPE surrounds both the nasal and temporal retina in only the most dorsal sections (Fig. 2A′–B′), whereas in middle and ventral sections only its temporal component is present (Fig. 2C′–E′). As both the nasal and temporal mutant RPE are present at E12.5 (Fig. 2F′–L′), the defect we observe at E11.5 reflects a developmental delay in the formation of nasal eye components.

In E12.5 control sections, *Sfrp2* labels all retinal cells in the proliferating layer and most of the retinal ganglion cells (RGCs) of the differentiating layer of the central retina (Fig. 2F–I) (Leimeister et al., 1998). However, *Sfrp2* staining is not observed in the CM (Fig. 2G,H and Liu et al., 2003). Consecutive dorsal-to-ventral *Foxg1*^−*/*−^ mutant sections labelled with *Sfrp2* reveal that, unlike the control, the mutant optic cup is not spherical (Fig. 2G′–L′). In addition, the opening of the optic cup containing the lens is only visible in the most ventral sections (Fig. 2J′,K′). This may be a consequence of the retina not growing properly along the nasal-temporal axis (the axis perpendicular to the optic stalk in controls), due to failure of the mutant optic fissure to seal.

**Suppl Fig. 1:**
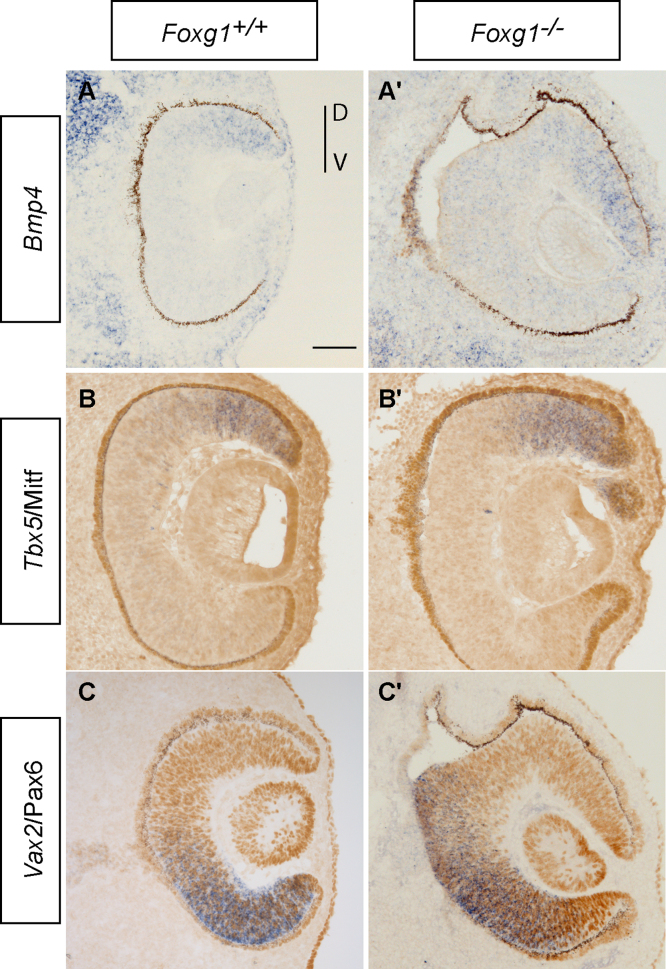
**Markers of dorso-ventral retina patterning show normal expression in the*****Foxg1***^***-/-***^**mutant.** In situ hybridisation for *Bmp4* (A,A′), *Tbx5* (B,B′) and *Vax2* (C,C′) on E12.5 control (*Foxg1*^*+/+*^) (A–C) and mutant (*Foxg1*^*-/-*^) retinal sections (A′–C′). In situ hybridizations for *Tbx5* and *Vax2* are followed by immunohistochemistry for Mitf and Pax6 respectively (brown staining in (B–C′). Scale bar in (A) corresponds to 100 μm and applies to all panels.

To study whether the abnormal *Foxg1*^−*/*−^ retina shows defects in patterning, we studied expression of *Bmp4* and *Tbx5*, which are normally found in the dorsal optic cup, and *Vax2*, which is normally expressed ventrally (Behesti et al., 2006). Our results did not reveal any significant differences between controls and *Foxg1*^−*/*−^ mutants in the patterns of expression of these markers (Suppl Fig. 1), in agreement with previous findings using other patterning molecules in these mutants (Pratt et al., 2004).

In summary, by E12.5, the defects observed at E11.5 become accentuated, with an obvious distortion of the normal spherical morphology of the mutant optic cup. At this stage, abnormal retinal folds are clearly detected nasally (Fig. 2G′–I′).

**Fig. 3:**
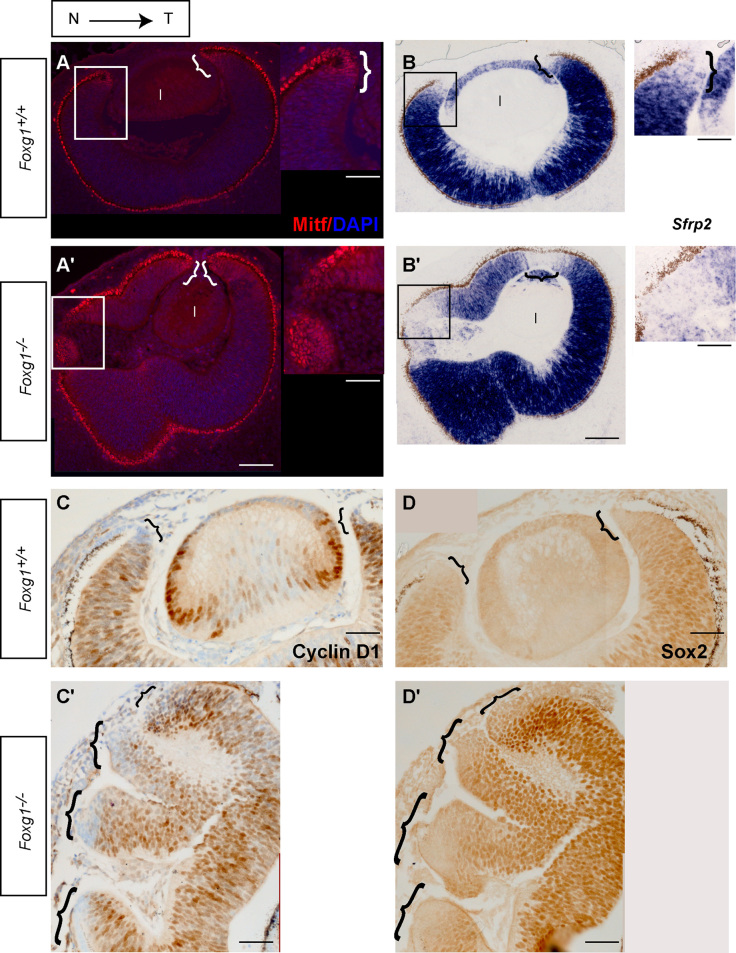
Additional ciliary margin (CM) tissue in *Foxg1*^−*/*−^E12.5 mutants. Immunofluorescence for Mitf in controls (*Foxg1*^*+/+*^) (A) and mutants (*Foxg1*^−*/*−^) (A′). In situ hybridisation for *Sfrp2* in controls (B) and mutants (B′). Immunohistochemistry for Cyclin D1 in controls (C) and mutants (C′) and for Sox2 in controls (D) and mutants (D′). Images (A–A′) are counterstained with DAPI (blue staining) and (C–C′) with cresyl violet. Sections (A,A′) are adjacent to sections (B,B′) respectively and (C,C′) to (D,D′) respectively. Squared areas in panels (A–B′) are depicted in a high power image to the right of each panel. Curly brackets indicate ciliary margin sites. Scale bars: (A–B′) low power images, 100 μm; high power images, 50 μm; (C–D′), 100 μm. (For interpretation of the references to colour in this figure legend, the reader is referred to the web version of this article.)

In addition to the above general morphological differences between controls and *Foxg1*^−*/*−^ mutants in the shape of the developing optic cup and retina, *Sfrp2* in situ hybridisation at E12.5 revealed additional *Sfrp2*-negative areas in mutants (compare Fig. 2G,H to H′-K′ and Fig. 3 below). These *Sfrp2*-negative areas are located predominantly nasally (see bracket and dashed line in Fig. 2H′), where *Foxg1* expression is normally highest.

### Ciliary margin tissue expansion in the *Foxg1*^−*/*−^ mutant

At E12.5, several molecules such as *Otx1*, *Mitf* and *Wnt2b* are expressed in the peripheral but not the central retina (Amae et al., 1998; Horsford et al., 2005; Liu et al., 2003; Martinez-Morales et al., 2001; Trimarchi et al., 2009). To confirm the presence of additional CM tissue in the *Foxg1*^−*/*−^ retina, suggested by the expanded *Sfrp2*-negative staining (Fig. 2), we examined expression of Mitf protein in E12.5 sections and compared it to the *Sfrp2*-negative domain.

In control sections, Mitf is specifically expressed in the RPE and at the tips of the CM (Fig. 3A and Amae et al., 1998; Horsford et al., 2005), in a region of the retina which does not express *Sfrp2* (compare positive staining in Fig. 3A with negative stained regions in Fig. 3B). In the *Foxg1*^−*/*−^ retina, expression of Mitf is also detected in the RPE and the tips of the CM as in controls (Fig. 3A′). However, the domains of Mitf expression are increased (Fig. 3A′) and coincide with the additional *Sfrp2*-negative regions (compare positive staining in Fig. 3A′ with negative stained regions in Fig. 3B′). Mitf staining in the *Foxg1*^−*/*−^ RPE appears normal.

To provide additional evidence of the expansion of CM tissue in the *Foxg1*^−*/*−^ retina, we examined expression of Cyclin D1 and Sox2 (Fig. 3C–D′), two proteins which are both normally absent from the CM region (Liu et al., 2007; Matsushima et al., 2011; Taranova et al., 2006). Immunohistochemistry for these markers revealed an expansion in both the Cyclin D1-negative (Fig. 3C′) and Sox2-negative (Fig. 3D′) domains of expression in the *Foxg1*^−*/*−^ mutant, in agreement with the results observed with *Sfrp2* (Fig. 3B′).

**Suppl Fig. 2:**
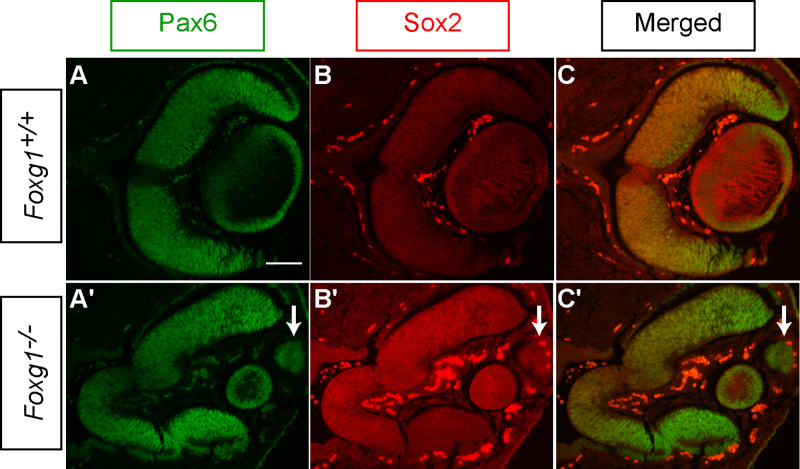
**Sox2 and Pax6 expression patterns are in agreement with an enlarged CM in the*****Foxg1***^***-/-***^**mutant.** Immunofluorescence for Pax6 (green) and Sox2 (red) on E12.5 wild type (A–C) and *Foxg1*^*-/-*^ mutant (A′–C′) horizontal sections. The arrow indicates a Pax6-positive;Sox2-negative area in the peripheral retina. Scale bar in (A) corresponds to 100 μm and applies to all panels.

Sox2 displays an inverse pattern of expression in the developing optic cup to that of Pax6 protein, with high expression of Sox2 in the central and of Pax6 in the peripheral retina (Matsushima et al., 2011). Double immunofluorescence for these proteins at E12.5, shows an expansion in the Pax6-highly-expressing; Sox2-negative domain of expression in *Foxg1*^−*/*−^ mutants (Suppl Fig. 2A′–C′) compared to controls (Suppl Fig. 2A–C), further confirming that the enlarged Sox2-negative domain in mutants corresponds to CM-like tissue.

**Fig. 4:**
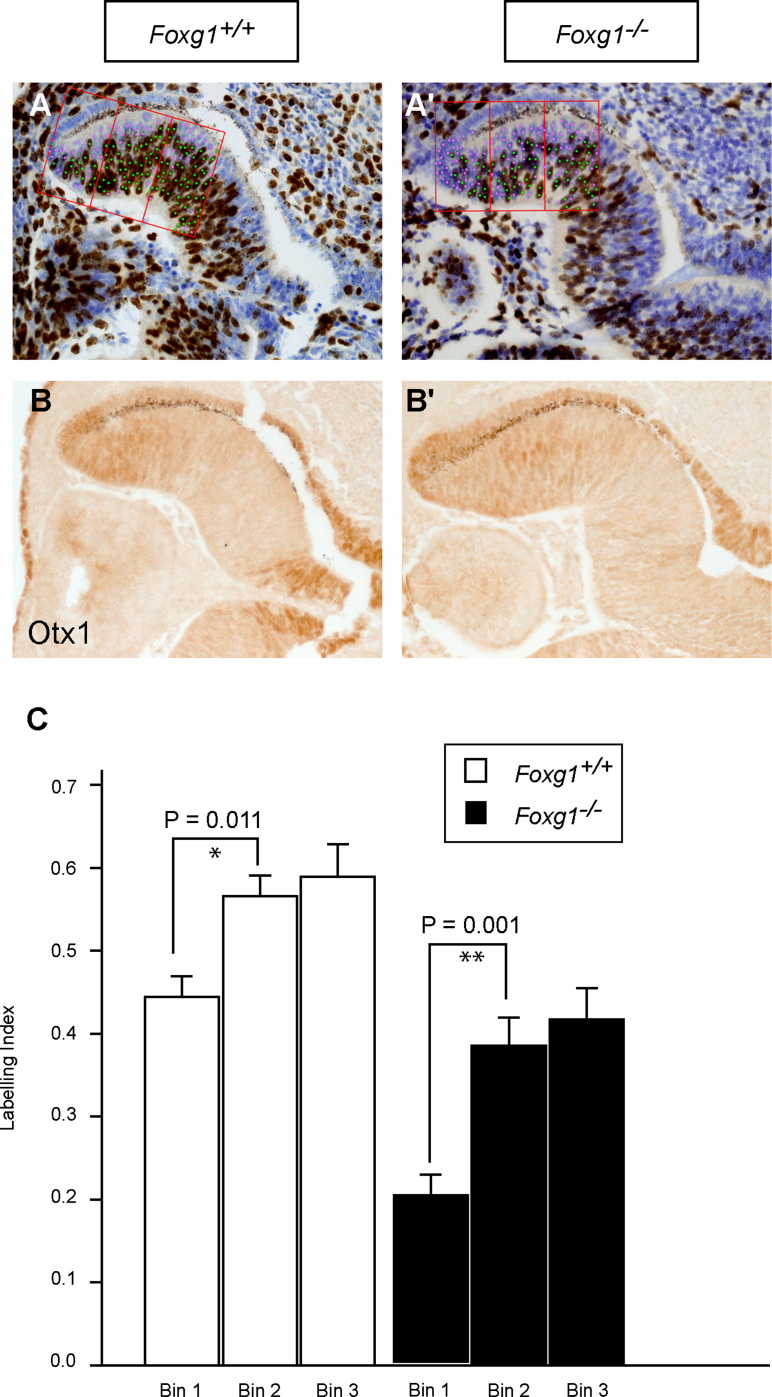
The cell cycle properties of the *Foxg1*^−*/*−^ peripheral retina resemble those of CM-like tissue. Immunohistochemistry for BrdU (A,A′) and Otx1 (B,B′) in E11.5 control (*Foxg1*^*+/+*^) (A,B) and mutant (*Foxg1*^−*/*−^) (A′,B′) horizontal retinal sections. Sections in (A,A′) are counterstained with cresyl violet. The counting bins used to calculate the labelling indices are shown in red; green dots represent the labelled BrdU-positive cells and pink dots the unlabelled cells (cresyl violet-positive) (A,A′). The labelling indices (fraction of BrdU-positive cells during a 30 min period) are plotted as average values from at least 3 different wild type (*Foxg1*^*+/+*^*)* and mutant (*Foxg1*^−*/*−^) retinas (C). The labelling indices were calculated within three consecutive counting bins in the peripheral retina (Bin 1 corresponds to the most peripheral bin). Error bars indicate standard deviation. (For interpretation of the references to colour in this figure legend, the reader is referred to the web version of this article.)

It has been shown that the peripheral retina has lower proliferation rates compared to the central retina (Fischer and Reh, 2000; Kubota et al., 2004; Liu et al., 2007). To substantiate that the expanded region in *Foxg1*^−*/*−^ mutants corresponds to CM-like tissue not only in terms of marker expression but also in terms of cellular behaviour, we examined the BrdU labelling index (LI) of the peripheral retinal region in controls and mutants. We performed this study at E11.5, when the morphological differences between *Foxg1*^−*/*−^ and control retinas are not as severe as those observed at later developmental stages (Fig. 4). We performed cell counts on serial sections (Fig. 4A,A′), using three consecutive cell counting bins placed at the peripheral retina, which was immunopositive for the CM marker Otx1 (Fig. 4B,B′). Our results revealed that in both control and *Foxg1*^−*/*−^ mutant sections the LI of bin 1 (most peripheral) was significantly different to the LI of bins 2 and 3 (Fig. 4C). In addition, the LI of *Foxg1*^−*/*−^ mutants was significantly reduced compared to that of controls in all areas studied (Fig. 4C), in agreement with the role of Foxg1 as a regulator of cell proliferation (Martynoga et al., 2005; Xuan et al., 1995). These results confirm that the peripheral retina in *Foxg1*^−*/*−^ mutants, as defined by expression of Otx1, shows cell cycle properties of CM-like tissue.

**Fig. 5:**
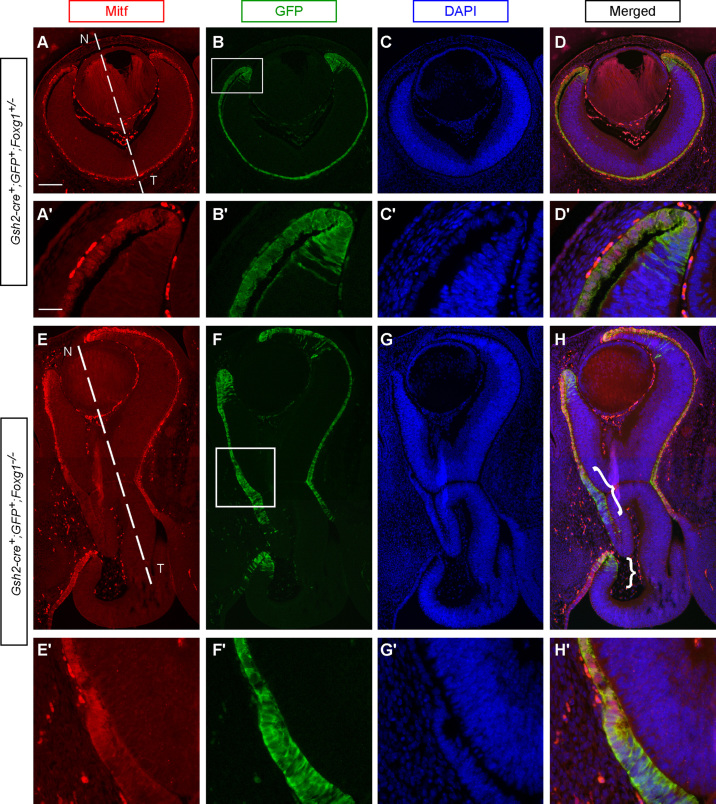
Evidence for additional ciliary margin (CM) sites in E14.5 *Foxg1*^−*/*−^ retinas. Immunofluorescence for Mitf (red) (A,A′,E,E′) and GFP (green) (B,B′,F,F′) in E14.5 horizontal retinal sections. Mitf is expressed in the retinal pigment epithelium (RPE) and in the most anterior tip of the control CM (A,A′). Strong GFP staining is found in the RPE and the entire CM of controls (B,B′). GFP and Mitf overlap in the RPE and partially overlap in the control CM (D,D′). In the null *Foxg1*^−*/*−^ retina there is Mitf staining in the RPE and the tips of the CM (E,E′) and intense GFP staining in the RPE and the entire CM (F,F′). Additional sites of GFP expression are found near the retinal folds (curly brackets in H,H′). These GFP-positive sites only partially express Mitf (E,E′). (C,C′) and (G,G′) are the control and mutant sections respectively counterstained with DAPI (blue). Panels (A′–D′) and (E′–H′) are high power images of the squared areas in (B) and (F) respectively. The dashed lines in (A) and (E) indicate the nasal (N) – temporal (T) divide in the control and the *Foxg1*^−*/*−^ mutant respectively. Scale bar in (A) corresponds to 200 μm and applies for panels (A–D) and (E–H). Scale bar in (A′) corresponds to 50 μm and applies for panels (A′–D′) and (E′–H′).

After E12.5 the *Foxg1*^−*/*−^ mutant retina begins to elongate. We examined how the CM region develops in these mutants at E14.5, using Mitf, which labels the RPE and the tip of the CM in control embryos (Fig. 5A,A′). We took advantage of a floxed-GFP reporter allele (Sousa et al., 2009; see Material and Methods for details) and a *Gsh2*^*Cre*^ allele (Kessaris, et al., 2006) whose combination labels the RPE and the entire CM region of controls at E14.5 (Fig. 5B,B′). GFP expression coincides with that of Mitf in the RPE and at the most distal tip of the CM (Fig. 5A–D, A′–D′). However, GFP also labels the rest of the CM, which is negative for Mitf expression, thus allowing us to distinguish between RPE and entire CM tissue. In the *Foxg1*^−*/*−^ mutants, Mitf is found in the RPE and at the edges of the peripheral retina (Fig. 5E). Similar to controls, GFP expression is also observed in the RPE and in the most peripheral retina that surrounds the lens, which corresponds to CM (Fig. 5F). However, GFP-positive cells are also observed at the edges of the folded retina (Fig. 5F–H, F′–H′; curly brackets in Fig. 5H). These GFP-positive sites express Mitf only at their tips, while the rest of the GFP-positive area is Mitf-negative (Fig. 5E,E′,H,H′), indicating that they correspond to CM, rather than RPE.

**Fig. 6:**
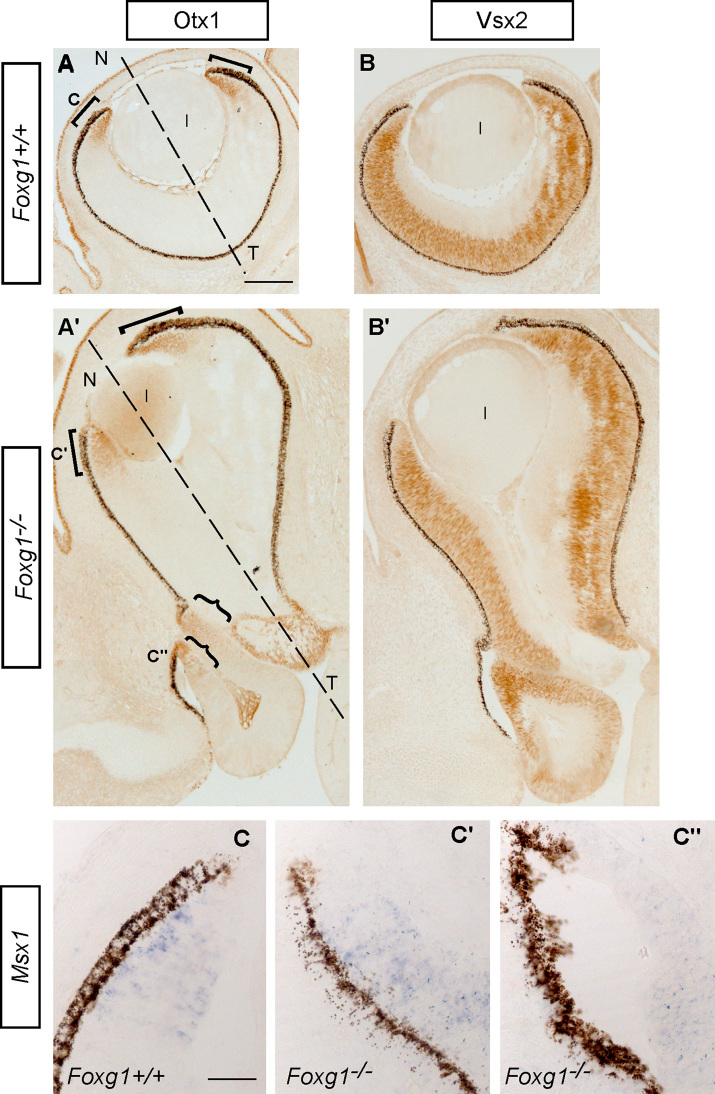
Additional ciliary margin (CM) tissue in E14.5 *Foxg1*^−*/*−^ retinas. Otx1 protein expression in the ciliary margin (CM) of control (*Foxg1*^*+/+*^) (A) and mutant (*Foxg1*^−*/*−^) (A′) retinas, indicated by brackets. In *Foxg1*^−*/*−^ mutants, additional sites of Otx1 expression surround retinal folds (curly brackets in A′). Immunostaining for Vsx2 in controls (B) and mutants (B′) revealing normal retinal progenitor proliferation, even in the mutant folds. *Msx1* mRNA expression in the CM of control and mutant retinas is in agreement with that of Otx1. *Msx1* is found in the CM margin of controls (C) and mutants (C′) and in additional sites in mutants (C′′). The *Msx1* positive sites correspond to the indicated sites in panels A (for C) and A′ (for C′, and C′′). The dashed lines in (A) and (A′) indicate the nasal (N) – temporal (T) divide in the control and the *Foxg1*^−*/*−^ mutant respectively. Abbreviations: l, lens. Scale bar in (A) corresponds to 200 μm and applies for panels (A–B′). Scale bar in (C) corresponds to 25 μm and applies for panels (C–C′′).

These results are further confirmed using Otx1, which strongly labels the CM region throughout development (Fig. 6A,A′ and Martinez-Morales et al., 2001; Trimarchi et al., 2009) and Vsx2, a marker of retinal progenitors (Fig. 6B,B′ and Liu et al., 1994). In *Foxg1*^−*/*−^ mutants, Otx1 expression was observed at the distal edges of the retina that flank the lens with a clear CM identity (Fig. 6A′). In addition, Otx1 expression was found in sites surrounding retinal folds (curly brackets in Fig. 6A′), in similar positions to those where GFP-positive cells were located (compare Figs. 5H′ to 6A′). Vsx2 was expressed in the proliferative retinal layer in *Foxg1*^−*/*−^ mutants (Fig. 6B′) as in controls (Fig. 6B) and expression continued into the folds confirming their retinal identity (Fig. 6B′).

*Msx1*, another CM marker (Monaghan et al., 1991) was found in the CM of controls (Fig. 6C) and *Foxg1* ^−*/*−^ mutants (Fig. 6C′), as well as in sites located nasally in the mutant, away from the discernible CM (Fig. 6C′′), in a similar fashion to that observed with Otx1 staining (compare Fig. 6C with the Otx1-positive region indicated as C in Fig. 6A and C′ and C′′ with the sites indicated with the same letters in Fig. 6A′.)

The above data collectively show that already at E12.5 there is an abnormal expansion of CM tissue in the nasal retina of *Foxg1*^−*/*−^ nulls and this expansion becomes more pronounced at later stages of development.

### Upregulation of Wnt/β-catenin signalling in the nasal CM of *Foxg1*^−*/*−^ embryos

Wnt2b and the canonical Wnt pathway have been implicated in the development of the CM in both chicks and mice (Cho and Cepko, 2006; Esteve et al., 2011; Fuhrmann et al., 2009; Kubo et al., 2003; Liu et al., 2003, 2006, 2007). We tested the hypothesis that Foxg1 normally suppresses the Wnt/β-catenin pathway. For this, we used E12.5 compound mutant embryos for *Foxg1*^−*/*−^ and the BAT-gal reporter, which carries β-gal under a TCF/LEF promoter and reports on Wnt/β-catenin signalling (Fotaki et al., 2011; Maretto et al., 2003) (*BAT-gal*^*+*^*;Foxg1*^−*/*−^ mutants and control littermates *BAT-gal*^*+*^*;Foxg1*^*+/+*^ or *BAT-gal*^*+*^*;Foxg1*^*+/*−^).

We first examined how expression of *Wnt2b* and that of the downstream Wnt/β-catenin signalling target *Lef1*, shown to be expressed in the peripheral retina (Liu et al., 2003, 2006), differed between E12.5 controls and *BAT-gal*^*+*^*;Foxg1*^−*/*−^ mutants.

**Fig. 7:**
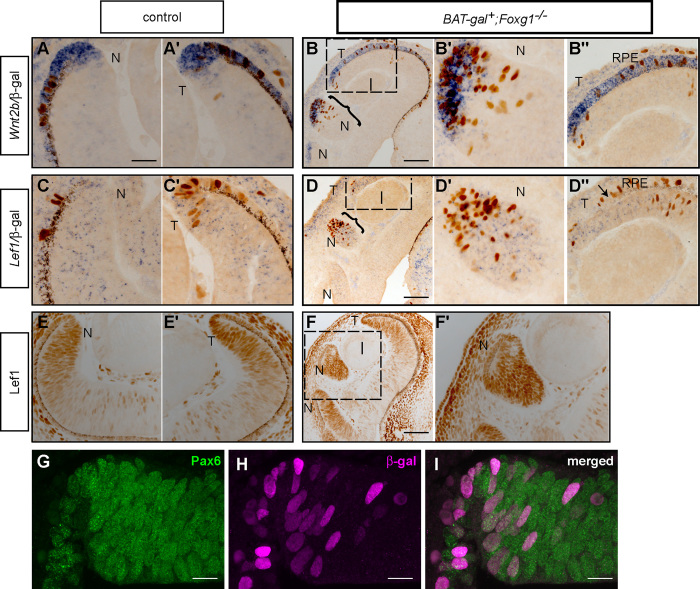
Upregulation of Wnt/β-catenin signalling in the nasal ciliary margin (CM) of E12.5 *BAT-gal*^*+*^; *Foxg1*^−*/*−^ compound mutants. Horizontal E12.5 retinal sections were studied by in situ hybridisation for *Wnt2b* (A–B′′) and *Lef1* (C–D′′) followed by immunohistochemistry for β-galactosidase (β-gal) in controls (A,A′,C,C′) and compound mutants (B–B′′and D–D′′). Immunohistochemistry for Lef1 in controls (E,E′) and *Foxg1*^−*/*−^ mutants (F–F′). Panels (A,B′,C,D′,E,F′) correspond to the nasal (N) and (A′,B′′,C′,D′′,E′) to the temporal (T) side of the same eye. The curly brackets in (B) and (D) indicate the nasal retinal folds shown in high power in panels (B′) and (D′) respectively, while the dotted squares indicated the temporal retina shown in high power in panels (B′′) and (D′′) respectively. The arrow in (D′′) indicates the start of the retinal pigment epithelium (RPE) in the temporal mutant retina. The dotted square in (F) delineates the high power image of the nasal folded retina in (F′). Immunofluorescence for Pax6 (green) (G), β-galactosidase (magenta) (H) and Pax6/β-galactosidase (I) on E12.5 *BAT-gal*^*+*^; *Foxg1*^−*/*−^ compound mutant retinas. Scale bar in (A) corresponds to 50 μm and applies for panels (A,A′,B′,B′′,C,C′,D′,D′′,E,E′,F′); (B,D,F), 100 μm; (G–I), 20 μm. Abbreviations: l, lens.

In controls, *Wnt2b* expression is detected in the RPE and is restricted to the tips of the CM in both the nasal (Fig. 7A) and temporal retina (Fig. 7A′). As with controls, *Wnt2b* is detected in the RPE and both the nasal (Fig. 7B,B′) and temporal CM of mutants (Fig. 7B′′). However, *Wnt2b* is not only detected in the nasal tip of the CM (Fig. 7B) but it is also strongly detected in the retinal folds, which form nasally (curly bracket in Fig. 7B, B′). Expression in the temporal tip of the CM seems to expand compared to controls (compare Fig. 7A′ with Fig. 7B′′).

In contrast to *Wnt2b*, *Lef1* expression is not restricted at the tip of the control CM, but extends throughout the peripheral retina both nasally (Fig. 7C) and temporally (Fig. 7C′), in a speckled fashion, consisting of expressing and non-expressing cells. *Lef1* expression in mutants is observed in the nasal (Fig. 7D,D′) and temporal (Fig. 7D′′) CM. Similar to controls, *Lef1* expression in *Foxg1*^−*/*−^ retinas displays a broader domain to that of *Wnt2b*. This is clearly appreciated in the folded areas, where *Wnt2b* is restricted at the periphery (Fig. 7B′), while *Lef1* is observed throughout the fold (Fig. 7D′).

**Fig. 8:**
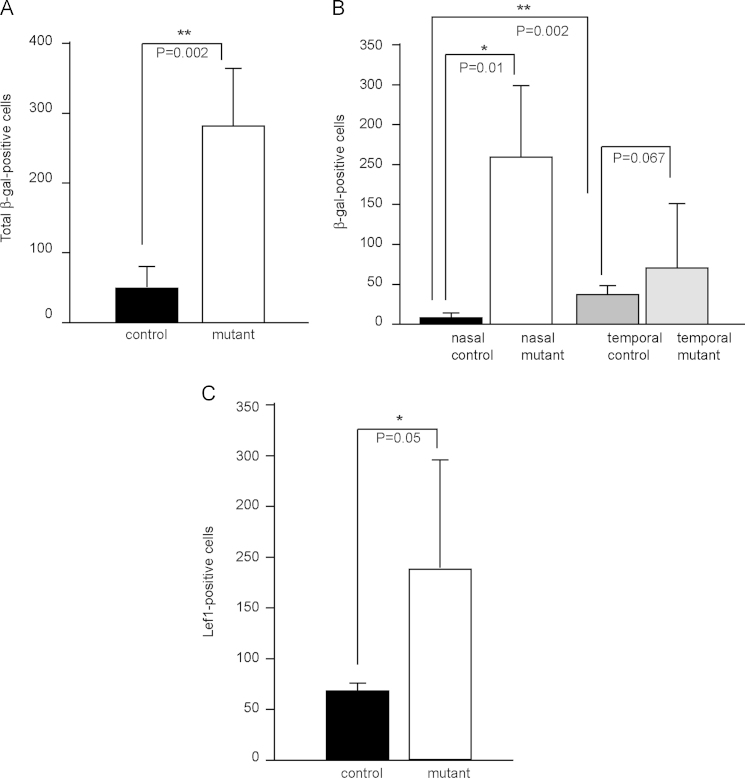
**Cell counts for immunopositive cells for downstream targets of the Wnt/**β**-catenin pathway.** Counts for total number of β-gal-positive cells (A) and β-gal-positive cells in the nasal and temporal retina (B). Counts for Lef1-positive cells in the nasal retina (C). Bars represent the median value of cell counts from at least 3 different retinas per genotype (Mann–Whitney test). Error bars indicate standard deviation.

We then examined the number and distribution of Wnt/β-catenin responsive cells, by performing immunohistochemistry for β-gal on the same control and mutant sections used for the in situ hybridizations described above. The total number of β-gal-positive cells in temporal and nasal CM combined showed a 5-fold increase in the mutants compared to controls (Fig. 8A). In controls, the majority of β-gal-positive cells detected were located in the temporal CM, either within the tips of the CM (*Wnt2b*- and *Lef1*-positive domains) or the broader CM area (*Wnt2b*-negative, *Lef1*-positive domains), with only very few detected nasally (compare Fig. 7A and C to A′ and C′ respectively and black bar to dark grey bar in Fig. 8B). In mutants, although the number of β-gal-positive cells in the temporal CM was higher than in controls, the difference did not reach statistical significance (compare Fig. 7A′ to B′′, C′ to D′′, and dark grey to light grey bar in Fig. 8B). However, the number of mutant β-gal-positive cells in nasal CM was 46-fold higher compared to controls (compare Fig. 7A to B′, C to D′ and black bar to white bar in Fig. 8B). Both nasally and temporally, mutant β-gal-positive cells were found within a *Wnt2b*-, *Lef1*-positive domain or a *Wnt2b*-negative, *Lef1*-positive domain (Fig. 7B–B′′ and D–D′′).

A similar significant increase in β-gal-positive cells was also observed in E14.5 *BAT-gal*^*+*^*;Foxg1*^−*/*−^ mutants (data not shown). As with the E12.5 sections this increase in mutants was attributed to an increase in nasally located CM cells. The majority of β-gal-positive cells for both groups was located in the most dorsal sections of the retina (not shown).

To corroborate the upregulation of Wnt/β-catenin signalling in the *Foxg1*^−*/*−^ mutant retinas, we performed immunohistochemistry for Lef1, a downstream target of this pathway. Lef1 protein was detected at high levels in the nasal and temporal CM of controls (Fig. 7E,E′) and *Foxg1*^−*/*−^ mutants (Fig. 7F) and in the nasal folds of mutants (Fig. 7F′). Cell counts of Lef1 immunopositive cells revealed a significant increase in the number of labelled cells in the nasal retina in mutants compared to controls (Fig. 8C), in agreement with the results obtained with β-gal.

It has previously been reported that upregulation of Wnt/β-catenin signalling in the mouse retina resulted in an increase in CM cell fates at the expense of NR fates. However, in that study the abnormally expanded CM region failed to express Pax6 (Liu et al., 2007), which is normally expressed by all cells in the retina, including the CM (Davis-Silberman et al., 2005). To examine whether the increased β-gal-positive cells in the compound *BAT-gal*^*+*^*;Foxg1*^−*/*−^ mutants also fail to express Pax6, we performed double immunofluorescence for these two proteins. Confocal microscopy revealed that in our model of Wnt/β-catenin upregulation, all CM β-gal-positive cells also express Pax6 (Fig. 7G I).

In summary, our results show an increase in Wnt/β-catenin signalling in the *Foxg1*^−*/*−^ CM, which is more pronounced in the nasal peripheral retina.

## Discussion

### Nasal retinal malformations in the *Foxg1*^−*/*−^ mutant

It has been shown previously that the *Foxg1*^−*/*−^ mutant eye displays severe defects in its most ventral components, where Foxg1 is strongly expressed early in development. The optic stalk fails to form and the eye joins to the brain via an elongated retina. In addition, the choroid fissure does not form ventrally, leading to a large ventral coloboma (Huh et al., 1999). Here, we characterised in detail how loss of Foxg1 affects the retinal phenotype of *Foxg1*^−*/*−^ mutants. We observed that, starting at E11.5, retinal tissue expands progressively and forms folds that become very pronounced by E14.5. These defects are prominent in the nasal retina where levels of *Foxg1* expression are normally highest.

### Expanded tissue in the *Foxg1*^−*/*−^ mutants has CM character

**Fig. 9:**
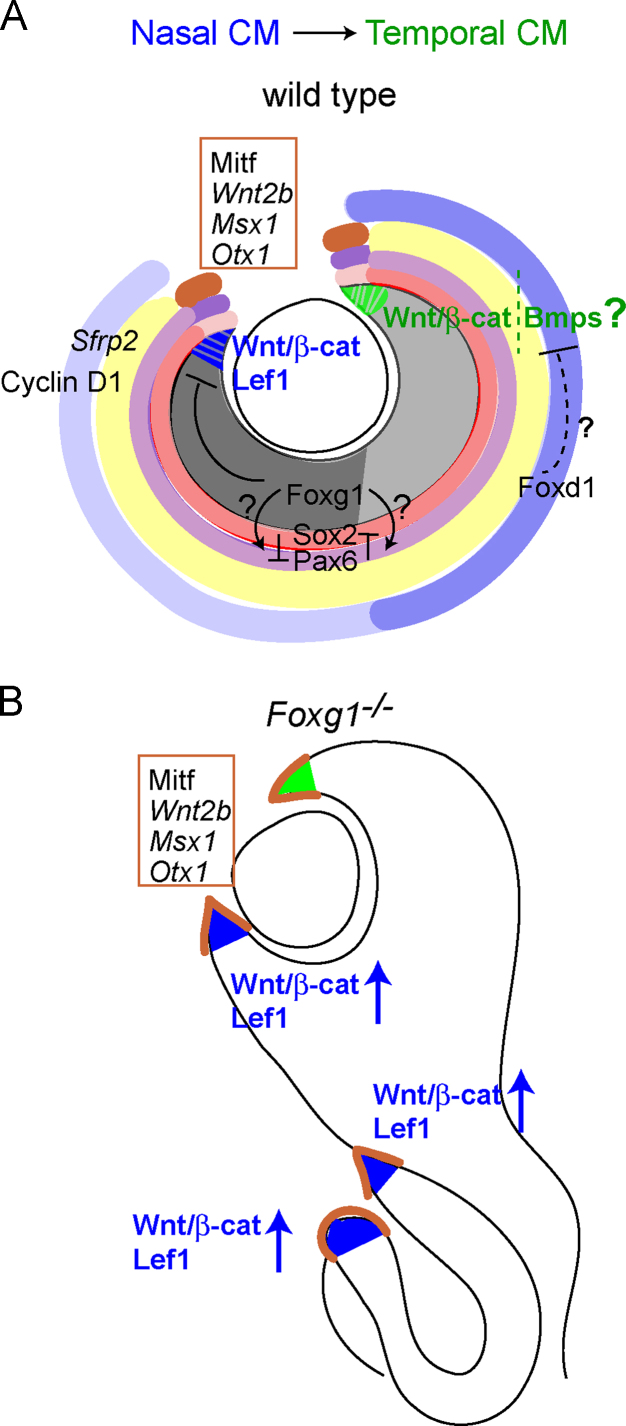
**Schematic diagram of the proposed role of Foxg1 in CM development.** Graded expression of *Foxg1* in the retina is shown in grey with a strong grey shade in the nasal and a lighter one in the temporal component of a wild type (*Foxg1*^*+/+*^) where Foxg1 normally represses the Wnt/β-catenin signalling pathway in the nasal CM (A). Specification of the temporal CM may require the Wnt/β-catenin and/or the Bmp signalling pathways. Foxd1, depicted in graded blue, may restrict expansion of temporal CM fates (A). The high-to-low gradient of expression of Sox2 and that of low-to-high of Pax6 from the central to the peripheral retina are shown in shades of red and purple respectively (A). The yellow shade depicts expression of the central retina markers *Sfrp2* and Cyclin D1 examined in this study (A). The CM markers studied are represented in brown in both the wild type (A) and mutant (*Foxg1*^−*/*−^) (B). In the mutant, Wnt/β-catenin signalling is upregulated and the nasal retina is expanded abnormally (B). (For interpretation of the references to colour in this figure legend, the reader is referred to the web version of this article.)

By studying the expression of several molecular markers we concluded that much of the nasally expanded retinal tissue in the *Foxg1*^−*/*−^ mutant has CM identity. The markers used included *Sfrp2*, Sox2 and Cyclin D1, which are all expressed in the entire retina except for the CM (Liu et al., 2007, 2003; Matsushima et al., 2011; Taranova et al., 2006), the CM markers Mitf (Horsford et al., 2005), Otx1 (Martinez-Morales et al., 2001; Trimarchi et al., 2009), *Msx1* (Monaghan, et al., 1991) and GFP expressed in CM at E14.5 following activation of a GFP-reporter by Gsh2-cre (Kessaris et al., 2006). Previous work described expansion of the CM in *Sox2*^−*/*−^ mutant mice (Matsushima et al., 2011). Inverse gradients of expression of Sox2 in the central retina and Pax6 in the periphery normally establish the boundary between the neurogenic and non-neurogenic retinal components respectively. Genetic ablation of Sox2 in mice results in cell fate conversion of the neural retina to CM, indicating that Sox2 is required for neural competence in the retina (Matsushima et al., 2011). Our findings indicate that Foxg1 also contributes to the suppression of CM fates, with its greatest effects in regions where its expression is normally highest. Unlike the effect of losing Sox2, however, many cells of the optic cup retain their retinal phenotypes, even in the nasal regions of *Foxg1*^−*/*−^ mutants, as shown for example by expression of the retinal progenitor marker Vsx2. Foxg1 appears to play a more subtle role compared to that of Sox2, perhaps suppressing the likelihood that cells outside the normal CM will develop a CM identity. Such an action might explain why additional ectopic CM regions develop in *Foxg1*^−*/*−^ mutants, often at a distance from the normal site of the CM (Fig. 9).

### Foxg1 antagonises Wnt/β-catenin signalling in the developing retina and CM

It is well established that the Wnt/β-catenin pathway plays a crucial role in specifying peripheral fates of the eye in both chicks and mice (Cho and Cepko, 2006; Esteve et al., 2011; Fuhrmann et al., 2009; Kubo et al., 2003; Liu et al., 2006, 2007). In the chick retina, in vivo activation of the Wnt/β-catenin pathway induced the formation of ectopic structures with CM character, leading to loss of retinal identity. Conversely, in vivo inhibition of the pathway led to a reduction in the CM, highlighting the fact that loss-of-function yielded results complementary to gain-of-function (Cho and Cepko, 2006). Similar to the chick, a stabilized form of β-catenin in an in vivo mouse model led to increased CM development, an effect that was more pronounced nasally (Liu et al., 2007). In addition, conditional ablation of β-catenin in the peripheral retina reduced the size of the CM (Liu et al., 2007). In agreement with this, based on absence of CM fates and expansion of the neural retina in *Sfrp1*^−*/*−^*;Sfrp2*^−*/*−^ double mutants, it has recently been proposed that the secreted frizzled-related proteins Sfrp1 and Sfrp2 fine tune Wnt/β-catenin signalling and cooperate to establish the border between the peripheral and central neural retina (Esteve et al., 2011).

Considering the above, the expansion of CM sites in the *Foxg1*^−*/*−^ mutant retina may result from an upregulation of the Wnt/β-catenin pathway, particularly in the nasal retina where Foxg1 is normally most highly expressed as proposed in Fig. 9. Thus, an additional role of Foxg1 in the embryonic eye may normally be to restrict the development of the CM by suppressing this signalling pathway. This is further supported by the fact that Foxg1 has been shown to repress Wnt/β-catenin signalling in the developing zebrafish forebrain, setting the boundaries between dorsal and ventral telencephalon as well as telencephalon and hypothalamus (Danesin et al., 2009). Expression of *Lef1*, a downstream target of Wnt/β-catenin signalling expressed throughout the CM (Liu et al., 2006), was more abundant in the *Foxg1*^−*/*−^ peripheral retina than in that of controls, and was observed in nasally formed folds with CM identity.

Our compound *Foxg1*^−*/*−^ mutant embryos carrying a BAT-gal allele, which reports on Wnt/β-catenin signalling, allowed us to further characterise changes in this pathway and quantify our results. This Wnt-responsive model has been recently used to study in detail Wnt signalling in the neighbouring RPE, but not the retina (Fujimura et al., 2009; Westenskow et al., 2009). Similar reporters have been used to study Wnt/β-catenin signalling in the embryonic retina using X-gal staining (Fuhrmann et al., 2009; Liu et al., 2003, 2007). Because β-gal is targeted to the nucleus in the BAT-gal mouse (Maretto et al., 2003), we were able to quantify Wnt-responsive cells by immunohistochemistry. In contrast to previous reports (Fuhrmann et al., 2009; Liu et al., 2007), we did not observe any Wnt-responsive cells in control central retina. This may be the result of the BAT-gal mouse under-reporting on Wnt signalling or context-dependent discrepancies between the different reporter mice (Barolo, 2006; Westenskow et al., 2009).

With our experimental approach we detected significantly more Wnt-responsive cells in the temporal than in the nasal CM of controls, indicating that the Wnt/β-catenin pathway is normally more active temporally than nasally. This raises the possibility that this signalling cascade may not be the principal pathway that normally determines CM specification nasally, or that suppression of this pathway is necessary for normal nasal CM development. It also suggests that specification of the nasal and temporal CM is controlled by different molecular programmes. A plausible additional candidate for normal nasal CM specification is Bmp signalling, which has been implicated in ciliary body formation (Zhao et al., 2002) (Fig. 9).

Regarding the compound *BATgal*^*+*^*;Foxg1*^−*/*−^ mutants, we observed an upregulation in the number of Wnt/β-catenin responsive cells nasally, indicating that loss of Foxg1 has a striking effect on the development of the nasal CM. How does Foxg1 control CM development nasally? Our in situ hybridisation results demonstrate high levels of *Foxg1* expression in the nasal retina and CM and lower levels in the corresponding temporal structures of controls. Based on our experimental results we propose that, nasally, Foxg1 normally represses Wnt/β-catenin signalling, restricting CM expansion (Fig. 9). In the temporal CM, where low levels of Foxg1 are present, this pathway remains active. Our results also suggest that another molecule normally restricts CM size expansion in the temporal peripheral retina. A likely candidate is the transcription factor Foxd1. This is based on its expression in the ventrotemporal peripheral retina (Carreres et al., 2011) and the fact that in both mouse and chick, Foxd1 and Foxg1 act antagonistically to promote specification of the temporal and nasal molecular properties of the retina respectively, which are necessary for proper formation of RGC projections (Herrera et al., 2004; Takahashi et al., 2009, 2003; Tian et al., 2008) (Fig. 9).

Our in vivo model, summarised in Fig. 9, proposes that loss of function of Foxg1 leads to over-expression of the Wnt/β-catenin signalling pathway and to an increase in the size of CM tissue in the peripheral retina. Foxg1 is a transcriptional repressor (Marcal et al., 2005; Yao et al., 2001), but at present we can only speculate as to which component of the pathway it suppresses in the peripheral retina. Similar to *wnt8b*, a direct target of foxg1 in the zebrafish telencephalon (Danesin et al., 2009), Foxg1 may bind to *Wnt2b*, the distal promoter of which contains two binding forkhead domains for members of the FOX family of transcription factors (Katoh, 2009). However, a recent report has argued against Wnt2b being the modulator of Wnt/β-catenin signalling in the mouse peripheral retina and proposes for this role the combinatorial actions of Sfrp1 and Sfrp2 (Esteve et al., 2011).

Another possibility is that Foxg1 represses one (or more) of the downstream targets of the Wnt/β-catenin pathway expressed in the peripheral retina, such as *Lef1*, *Tcf1*, *Tcf3* and *Axin2* (Liu et al., 2006). To date there is no direct evidence for such transcriptional control. However, the fact that Foxg1 interacts with members of the Groucho/TLE family of co-repressors (Roth et al., 2010; Sonderegger and Vogt, 2003; Yao et al., 2001), which bind to TCF/LEF sites repressing Wnt/β-catenin signalling transcription (Levanon et al., 1998; Roose et al., 1998), raises the possibility that Foxg1 may also participate in this inhibitory complex. Foxg1 may also bind to β-catenin in the cytoplasm, where it is known to localise in differentiating cells (Regad et al., 2007), inhibiting its translocation to the nucleus and subsequent activation of downstream targets of the pathway.

## Conclusions

In this report, we examine the *Foxg1*^−*/*−^ retinal phenotype and find an expansion of CM tissue, which is more pronounced in nasal retina where *Foxg1* is normally expressed at high levels. Our Wnt/β-catenin signalling reporter mouse reveals that in controls this pathway is mainly active in the temporal CM, while in *Foxg1*^−*/*−^ mutants it is over-activated nasally. This suggests that Foxg1 restricts CM expansion nasally, while a molecule other than Foxg1 plays an analogous role in the temporal CM. Our data are in agreement with a new role of Foxg1 in restricting the development of the CM nasally and establishing the boundary between the neurogenic and the non-neurogenic retina.

## Funding source

The Wellcome Trust and MRC, UK.
